# Population genetic processes affecting the mode of selective sweeps and effective population size in influenza virus H3N2

**DOI:** 10.1186/s12862-016-0727-8

**Published:** 2016-08-03

**Authors:** Kangchon Kim, Yuseob Kim

**Affiliations:** 1 Interdisciplinary Program of EcoCreative, Ewha Womans University, Ewhayeodae-gil 52, Seodaemun-gu, Seoul 120-750 South Korea; 2 Department of Life Sciences, Ewha Womans University, Ewhayeodae-gil 52, Seodaemun-gu, Seoul 120-750 South Korea

**Keywords:** Influenza virus, Positive selection, Background selection, Metapopulation, Soft sweep

## Abstract

**Background:**

Human influenza virus A/H3N2 undergoes rapid adaptive evolution in response to host immunity. Positively selected amino acid substitutions have been detected mainly in the hemagglutinin (HA) segment. The genealogical tree of HA sequences sampled over several decades comprises a long trunk and short side branches, which indicates small effective population size. Various studies have reproduced this unique genealogical structure by modeling recurrent positive selection. However, it has not been clearly demonstrated whether recurrent selective sweeps alone can explain the limited level of genetic diversity observed in the HA of H3N2. In addition, the variation-reducing impacts of other evolutionary processes – background selection and complex demography – relative to that of positive selection have never been explicitly evaluated.

**Results:**

In this paper, using computer simulation of a viral population evolving under recurrent selective sweeps we demonstrate that positive selection alone, if it occurs at a rate estimated by previous studies, cannot lead to such a small effective population size. Genetic hitchhiking fails to completely wipe out pre-existing variation because soft, rather than hard, selective sweeps prevail under realistic parameters of mutation rate and population size. We find that antigenic-cluster-transition substitutions in HA occur as hard sweeps. This indicates that the effective population size under which those mutations arise must be much smaller than the actual population size due to other evolutionary forces before selective sweeps further reduce it. We thus examine the effects of background selection and metapopulation dynamics in reducing the effective population size, using parameter values that reproduce other aspects of molecular evolution in H3N2. When either process is incorporated in recurrent selective sweep simulation, selective sweeps are mostly hard and the observed level of synonymous diversity is obtained with large census population size.

**Conclusions:**

Background selection and metapopulation dynamics have greater variation reducing power than recurrent positive selection under realistic parameters in H3N2. Therefore, these evolutionary processes are likely to play crucial roles in reducing the effective population size of H3N2 viruses and thus explaining the characteristic shape of H3N2 genealogy.

**Electronic supplementary material:**

The online version of this article (doi:10.1186/s12862-016-0727-8) contains supplementary material, which is available to authorized users.

## Background

The global epidemics of human seasonal influenza are of great concern in public health. The analysis of viruses’ sequence diversity over time and space plays a key role in understanding the epidemiology, as the pattern of their transmission and interaction with host immunity may uniquely shape the trajectory of their evolutionary changes [1]. Among the strains of human influenza viruses, the subtype H3N2 of type A has been the most common cause of seasonal influenza and is also the best understood system evolutionarily due to a large number of gene sequences, among which the most numerous is of hemagglutinin (HA) gene segment, accumulated over several decades. Hemagglutinin, coded by one of 11 genes located on the eight segments of the viral RNA genome, is a surface glycoprotein that mediates the entry of the viral particle into the host cell and, along with neuraminidase (NA), is recognized as an antigen by the host immune system. Both HA and NA genes are observed to undergo rapid evolutionary changes in amino acid sequences [2]. Many of these amino acid substitutions are believed to alter the antigenic structure of the proteins, which allows viruses to evade host immunity and thus confers an evolutionary advantage to the variant. Clear evidences of positive selection on such variants were obtained when various methods for detecting positive selection were applied to influenza virus sequences [2–6].

An overview of the evolutionary history of influenza viruses is provided by a genealogical tree of viral sequences sampled over multiple influenza seasons. The construction of genealogy (or coalescent tree) is possible when phylogenetic inference is applied to a non-recombining portion of the genome. A characteristic structure of genealogical tree was recognized: it has a long “trunk”, which represents one successfully surviving viral lineage through time, and other short side branches which go extinct within a few years [1]. This tree topology simply indicates that the effective population size of seasonal viruses is small relative to the time-scale of serial sequence sampling [7]: the lineages of two randomly chosen sequences at a given time coalesce to a common ancestor only one or two years back in time [8, 9], as if there are only a small number of viruses in the entire global population at a given time. The definition of effective population size, *N*_e_, is the number of individuals in a hypothetical population that reproduces according to a standard discrete-generation model (the Wright-Fisher model), the basic evolutionary genetic property (e.g. the level of polymorphism or the rate of adaptation) of which is equivalent to that of the actual population of interest. For example, the expected heterozygosity at a nucleotide site is given by π = 2*N*_e_μ, where μ is per-generation mutation rate. Applying a well-estimated mutation rate per year and assuming 4.5 days per generation (see below), we obtain *N*_e_ on the order of 100 for H3N2 viruses. This effective size is in stark contrast to the actual number (census size) of H3N2 viruses. Considering the large number of human individuals infected with seasonal influenza viruses such as H3N2 at a given time, the census size of viral population is believed to be several orders of magnitude larger than the effective size [10].

This small effective size of the viral population is generally attributed to positive selection driven by host immunity. As it is known that homologous recombination within an influenza viral segment does not occur [11], when a sequence carrying a new beneficial allele - a nonsynonymous mutation causing antigenic change or drug resistance - sweeps through the population, it will wipe out pre-existing nucleotide diversity in the population. This effect is termed genetic hitchhiking or selective sweep [12]. Lower sequence diversity (faster coalescence) of the HA segment compared to other segments [8] is one of many evidences for recurrent selective sweeps operating in H3N2. Other evidences include the excess of nonsynonymous substitutions at epitope sites [3, 4] and various aspects of allele frequency changes compatible with clonal interference [5]. Clonal interference occurs when a high rate of positive selection results in beneficial alleles arising at multiple loci that compete with each other to cause the loss of less-strong beneficial alleles [13–15]. Whether the temporal clustering of mutant alleles’ fixation events observed in H3N2 viruses is a result of clonal interference was recently questioned because it was found that the stochastic fluctuation of genealogical processes in asexual population can generate such clustering [16]. However, in the same paper, recurrent positive selection was shown to be essential in explaining the observed level of clustering, which was actually significantly lower than expected under neutrality, in H3N2. Therefore, all of these studies strongly support the operation of recurrent positive selection, thus selective sweeps in the H3N2 population.

However, it was not demonstrated clearly whether selective sweeps alone can explain the limited level of sequence polymorphism observed in H3N2. One should first find out whether the rate of nucleotide substitutions driven by positive selection (henceforth called “adaptive substitutions”) in H3N2 is high enough to cause strong reduction of effective size and genetic diversity. There are however conflicting assessments regarding the rate of adaptive substitutions. Koelle et al. [17] proposed that antigenic cluster changes in H3N2 at 2-8 year intervals are caused by episodes of strong selection separated by periods of neutral evolution. A similar pattern of evolution was shown to emerge when the appearance of a new successful antigenic variant is mutation-limited under a simple model of inter-strain competition and cross-immunity [18]. Recent experiments showed that most of such antigenic cluster transitions can occur by single amino acid substitutions [19]. Other studies however found that such “episodic” sweeps cannot generate the observed genealogical structure and suggest that many nonsynonymous sites other than those responsible for major cluster transitions are under positive selection [5, 10]. Fortunately, the rate of adaptive substitutions may be estimated independently, without considering the rate of antigenic changes and the shape of genealogy, by applying an extension of the McDonald-Kreitman test [20, 21]: Bhatt et al. [2] estimated that about 1.3 adaptive substitutions per year occurs in the HA gene; thus, a higher rate than that considering only major antigenic substitutions. Strelkowa and Lässig [5] also obtained a similar estimate using an equivalent method. They find that this rate is high enough to cause frequent clonal interference [13, 14]. In this study, as we are interested in the maximum reduction of variation by recurrent selective sweeps alone, we use an upper estimate of adaptive substitution rate (at least 1.3 per year).

It is also important to examine whether selective sweeps in influenza viruses occur as hard versus soft sweeps. When a beneficial mutation that arose once by mutation reaches fixation, it will completely erase polymorphism that existed at the time of mutation (given complete linkage). This event is called a hard selective sweep and was considered in the initial studies of selective sweeps [12, 22]. On the other hand, if multiple copies of a beneficial mutation at a single nucleotide site that arose independently by recurrent mutation (thus identical by state) reach high frequencies together and replace the former wild-type allele, they cannot completely erase the previous polymorphism because multiple linked haplotypes hitchhike along these beneficial copies. This process is defined as a soft selective sweep [23]. The prevalence of soft sweeps is known to increase with population size multiplied by per-site mutation rate, because this product (scaled mutation rate) determines the opportunity for recurrent introduction of the same beneficial allele. Therefore, investigating which mode of selective sweeps operates in H3N2 is likely to inform us about the (effective) population size under which positive selection occurs and about the variation-reducing power of selection.

Other evolutionary genetic forces may also contribute to a limited level of sequence polymorphism. If negative selection removes deleterious mutations that arise very frequently on a sequence with little or no homologous recombination, its cumulative effect can greatly reduce the number of viral sequences being inherited and this effect is called background selection [24]. Previous work by Strelkowa and Lässig [5] revealed that most of nonsynonymous mutations occurring in influenza sequences are under negative selection and discussed that background selection interferes positively selected alleles. Łuksza and Lässig [9] demonstrated that deleterious mutational load is a key to determine the fitness of the viral lineages. However, the impact of background selection on sequence variation has not yet been explicitly estimated. Due to its high mutation rate and to there being no within-segment recombination, the effective population size of influenza virus may be significantly decreased by this process. The complex demographic dynamics of seasonal influenza virus is another factor that can significantly decrease its effective population size. Recent investigations demonstrated that global patterns of H3N2 influenza epidemics are explained by the complex seasonal and inter-regional migration/seeding of viruses [25, 26], mostly from Asia to the rest of the world [27, 28]. Such complex demographic dynamics of the global H3N2 population (metapopulation dynamics; seasonal extinction and recolonization of most local populations except for a potential reservoir from which the global spread is seeded) may imply recurrent population bottlenecks for the global H3N2 population, the effect of which on genetic variation would be similar to that of recurrent selective sweeps. The major aim of this study is to quantitatively evaluate the relative contributions of positive selection, negative selection, and complex demography in reducing the effective population size of H3N2 viruses. Using the simulation model of viral population that incorporates all of these evolutionary forces, we will conclude that background selection and metapopulation dynamics have much greater variation-reducing effect than recurrent selective sweeps.

Recently, Koelle and Rasmussen [29] reported their investigation on the effect of deleterious mutations on the pattern of molecular evolution in H3N2. They reached a conclusion similar to ours: recurrent negative selection limits the emergence of advantageous (new antigenic) variants and, together with selective sweeps, the overall size of genealogy. However, whereas they modeled the evolutionary process embedded in continuous-time susceptible-infected-recovered (SIR) epidemiological dynamics, our model uses a conventional population genetic approach using discrete generations, fixed (census) sizes of viral population, and effects quantified by neutral (synonymous) polymorphism. This makes it easier to interpret our results in comparison with those in population genetics literature and to quantitatively evaluate the relative effects of variation-reducing evolutionary genetic processes. Furthermore, as the evolutionary genetic processes at synonymous and nonsynonymous sites are explicitly modeled, our approach allows us to estimate the strengths of negative selection from the observed nonsynonymous-to-synonymous substitution ratio in H3N2.

## Results and discussion

### Low synonymous diversity of HA cannot be explained by positive selection alone

We investigated whether the variation-reducing effect of recurrent positive selection, the rate of which was recently inferred to be ~1.3 adaptive substitutions per year on the HA segment [2], is sufficiently strong to explain the observed level of synonymous diversity without other mechanisms known to reduce genetic variation. Synonymous diversity π_s_ of HA1 was estimated to be 6.2 (per segment) when we calculated mean pairwise synonymous substitutions among sequences sampled within the same year and obtained its average over 1968 to 2013. Then we performed a simulation of recurrent selective sweeps in a viral population that reproduces in discrete generations. Here, one generation corresponds to the duration of viral transmission from one host to another. This approach of modelling each infected host by a single representative gene copy, which is a fair approximation if superinfection is rare and the differential replication of alleles due to within-host dynamics (mutation and selection over multiple rounds of viral replication) is translated into the rate of transmission between hosts, was used in previous studies [10, 29, 30]. Therefore, the meaning of population size, mutation rate, and selection strength in this model needs to be interpreted accordingly. We assume that a single generation corresponds to 1/80 year which is approximately the estimated interval between host-to-host infection [31]. Then, to yield 8.0 × 10^−3^ substitutions per site per year by neutral genetic drift, which is approximately the synonymous substitution rate that we obtained from sequence divergence over 46 years, we set mutation rate to μ = 1.0 × 10^−4^ per site per generation. As our modelling focuses on the HA1 segment of H3N2, the length of a viral sequence is initially *L* = 1,000 (sites). Each sequence is made up of *L*_s_ = 230 “synonymous” sites, which is approximately the average number of synonymous sites at HA1, and *L*_n_ = *L* - *L*_s_ nonsynonymous sites. A subset of nonsynonymous sites (“beneficial sites”) mutate into advantageous alleles with selection coefficient *s* in the range of between 0.05 and 0.2 (see Methods for the choice of this range of *s*) at a rate μ_b_ = μ/3. At this point, mutations at the rest of nonsynonymous sites are assumed to be selectively neutral (Model A). For the given input parameters of positive selection (μ_b_ and *L*_b_), the rate of adaptive substitutions, *k*, would increase with increasing *s* and *N* (while the relationship between *s, N* and *k* is not straightforward due to clonal interference [14]). As we are primarily interested in examining genetic variation across different evolutionary conditions that nevertheless generate the same rate of adaptive substitutions, the value of *L*_b_ was adjusted by trial and error to yield *k* close to 1.3 or 2.0. During the simulation run, the fixation probability of each beneficial allele is much lower than 2 *s*, confirming that strong clonal interference is operating at this rate of adaptive substitutions [5].

**Fig. 1:**
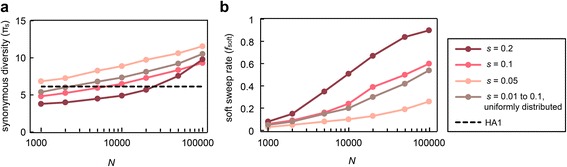
Simulation of recurrent positive selection (model A) under various population sizes and selection coefficients. A sequence of length *L* = 1,000 contains 230 synonymous sites over which the level of polymorphism is calculated, which models the HA1 segment of H3N2 virus. As we reduced *L*_b_ (the number of beneficial nonsynonymous sites) with increasing *s* and *N*, the numbers of adaptive substitutions per year are similar (*k* ≈ 1.3) in all cases. **a**. Per-segment synonymous diversity (π_s_) as a function of population size with different assumptions on selection coefficient *s* for beneficial mutations. π_s_ obtained from HA1 is indicated by a dashed line. **b**. The rate of soft sweeps (*f*_soft_) in the same simulation replicates

First, we ran a number of simulations with varying population size *N* (from 10^3^ to 10^5^) while keeping *k* close to 1.3 by adjusting *L*_b_ (Model A; Additional file 1: Table S1). Synonymous diversity, π_s_, increases as a function of *N* (Fig. 1a). For example, simulation with *s* = 0.1 and *N* = 5,000 generates π_s_ = 6.05, slightly lower than the observed value. With larger population sizes π_s_ below 6.2 is not observed. When selective sweeps are more frequent to yield *k* ≈ 2.0, π_s_ is lower than when *k* ≈ 1.3, as expected (Additional file 1: Table S1). However, synonymous diversity still increases monotonically with *N* and, for *s* = 0.1, exceeds the observed value as *N* increases over 20,000. We also find that, conditional on *k* and *N*, π_s_ increases with decreasing *s* (Fig. 1a). However, even with large *s* (0.2), π_s_ < 6.2 is satisfied only with *N* < 30,000. This result suggests that if the population size of H3N2 viruses is much larger than the order of 10^4^, which is likely to be true, the genetic hitchhiking caused by one or two adaptive substitutions per year cannot reduce genetic variation to the observed level unless there are other evolutionary forces to reduce the effective population size of H3N2.

The classical theory of genetic hitchhiking [22], which assumes hard selective sweeps (i.e. a beneficial allele of a single mutational origin sweeps at each substitution event), predicts that *k* imposes the upper bound of the level of neutral polymorphism: given sufficiently large *N* (> > 80/*k*), as polymorphism in the population is swept away (thus gene lineages coalesce) at a rate *k* per year on average, pairwise sequence diversity (π_s_) is predicted to be approximately $\left(\frac{2}{k}\right)80\mu {L}_{\mathrm{s}}$. (Branch lengths of coalescent tree are limited to ~1/*k* and therefore the size of genealogy for two sequences is ~2/*k*.) Predicted π_s_ is 2.83 with *k* = 1.3, while π_s_ in our simulation (Fig. 1a) far exceeds this value. Furthermore, the monotonic increase of π_s_ with increasing population size is not predicted either. There are at least three potential reasons for this discrepancy between the simple theory of hitchhiking and our result. First, recurrent fixations driven by positive selection may not occur independently but clustered in time due to stochastic whole-population coalescent processes [16] and clonal interference [5]. Namely, multiple beneficial alleles would often sweep to fixation jointly. Then, the expected waiting time (going backward) until two gene lineages coalesce could be longer than 1/*k*. Second, even though our simulations were performed conditional on the same number of adaptive substitutions per year, simulated populations of larger *N* must have experienced more intense clonal interference, and thus more beneficial alleles lost by competition. It is known that clonal interference effectively reduces the strength of positive selection [14]. Given that smaller *s* leads to larger π_s_ (Fig. 1a), an effective decrease in the strength of selection with increasing clonal interference may explain the monotonic increase of π_s_ with *N*. Third, the fixation of a beneficial allele at each site may not result in a hard sweep but a soft selective sweep [23] in our simulation. As we find that considering soft selective sweeps is the key to finding appropriate evolutionary models for H3N2 viruses, we analyze this effect in detail below.

### Rate of soft selective sweeps in H3N2 viruses

Genetic variation is incompletely wiped out by soft sweeps, which occur when multiple copies of a beneficial allele at a single nucleotide site that are identical in state but of different mutational origins, and that are thus linked to different neutral variants, contribute together to an episode of adaptive substitution. Note that our simulation permits soft sweeps as mutation at each site can occur recurrently. Soft selective sweep occurs with probability approximately proportional to scaled mutation rate θ = 2*N*μ [23]. Therefore, it can explain increasing synonymous diversity with *N* in our simulation. Here, *N* is the population size that determines opportunity for multiple recurrent mutations arising at a single site. If *N* for H3N2 population is very large, most adaptive substitutions would occur as soft sweeps. Therefore, estimating the rate of soft selective sweeps from the viral sequences would shed light on the (effective) population size under which viruses experience positive selection.

**Fig. 2:**
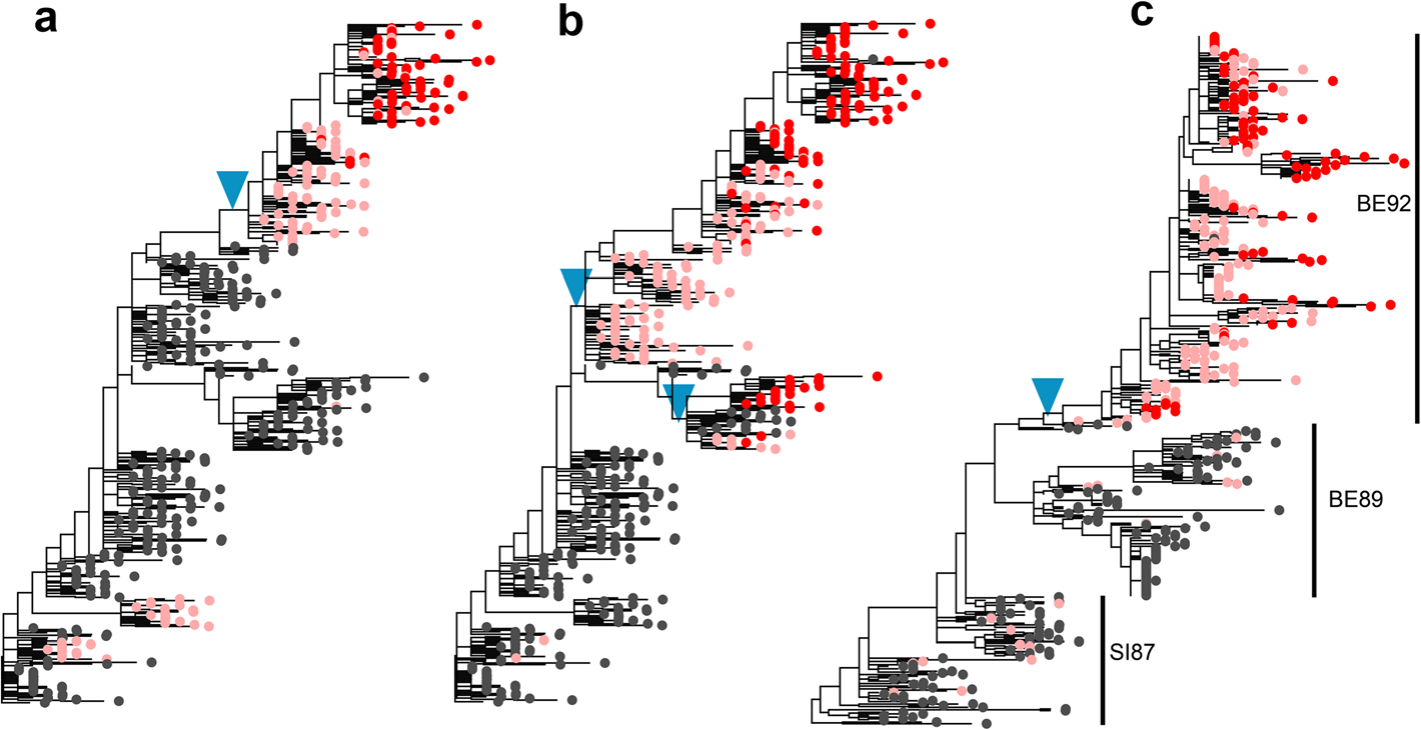
Distinguishing the mode of selective sweep on genealogy. A maximum likelihood tree is reconstructed for simulated (or actual HA segment) sequences sampled over a period that starts before the first mutational origin and ends after the fixation of a (putatively) beneficial allele. The copies of the beneficial allele at the branch tips are shown as *pink* or *red dots*. *Red dots* indicate copies observed when they reached fixation in the population. **a**. An example of hard selective sweep. All copies of the beneficial allele at the time of fixation (*red dots*) originate from a single mutation (indicated by *blue triangle*). **b**. An example of soft selective sweep. There are two clones originating from two different mutation events (two *blue triangles*) at the same site at the time of fixation. Examples A and B, from two different sites on the same genealogy, were obtained from the simulation of model B1 with *L* = 1,000 and *N* = 10,000. **c**. A hard selective sweep of the 156 K allele during antigenic cluster change from SI87 to BE92. Identical 156 K mutations arose independently (*pink*) and co-existed separately in BE89 and BE92 clusters. However, when allele frequency became 1.0 (*red*), only 156 K mutants in BE92 existed

We investigated the mode of selective sweeps of 17 antigenic-cluster-transition substitutions from 1968 to 2002 at seven amino acid positions near the receptor binding site [19]. These substitutions are very likely to be driven by positive selection, since their individual antigenic impacts were verified experimentally. For each substitution, a maximum likelihood tree was constructed using all the available sequences sampled from one year before the derived allele’s appearance to the year of its fixation. A selective sweep is soft if multiple copies of an identical beneficial allele arise independently from different ancestral backgrounds, which results in mapping of mutations on different branches of the genealogical tree, and reach fixation together (*i.e.* more than one clone carrying the beneficial allele persist when the frequency of the derived allele reaches 1.0, see Fig. 2a and b for illustration).

Interestingly, we do not find the pattern indicating a soft sweep in any of the 17 cases. In most cases, the patterns are compatible with hard sweeps. Namely, an antigen changing mutant is observed to occur in only one ancestral background and then to increase to high frequency. In some genealogies we observe different copies of the same beneficial allele arising separately in different backgrounds, but only one clone of the allele going to intermediate frequency and reaching fixation (see Fig. 2c for an example). An identical observation was reported by Strelkowa and Lässig [5]. In summary, the rate of soft sweeps on the HA gene in H3N2 population is very low.

To find out in what range of population size the rate of soft sweeps is as low as observed above, we examined the rate of soft sweeps in simulated datasets under models of positive selection considered above. We examined the number and frequency of clones originating from independent beneficial mutations and obtained the proportion, *f*_soft_, of simulated selective sweeps that generate the pattern of soft sweep (see Methods), while keeping the number of sampled sequences very similar to that of actual viral sequences. With *k* ≈ 1.3 and *s* = 0.1 (Model A), in order for *f*_soft_ to be as low as 0.1, for example, *N* should be as small as 2,000 (Fig. 1b). When selection is weaker with *s* = 0.05 and the number of beneficial sites (*L*_b_) is increased to yield the same rate of advantageous substitutions (see Additional file 1: Table S1 for *L*_b_), *f*_soft_ at *N* = 2,000 is lowered to 0.06. However, under this parameter set, synonymous diversity increases to 7.35 (Fig. 1a). Simulation results with other parameter values also show that, with recurrent positive selection alone, it is not possible to achieve both *f*_soft_ < 0.1 and π_s_ < 6.2 if *N* > = 10^4^ (Fig. 1, Additional file 1: Table S1).

### Contribution of background selection

The above results show that low level of synonymous (neutral) polymorphism and the prevalence of hard selective sweeps observed in H3N2 viruses cannot be explained unless assuming a small population size (< 10^4^) under which positive selection operates. As the actual size of the viral population is likely to be much larger than that, one needs to invoke the presence of other evolutionary forces that significantly reduce the effective population size (thus genetic variation) of H3N2 before positive selection further reduces it. The first candidate is the effect of negative selection against deleterious mutations (“background selection” [24]). Given that the majority of nonsynonymous sites in a viral genome are believed to be under negative (or purifying) selection [9, 32, 33], the absence of intra-segment homologous recombination and limited reassortment, recombination between different segments (which requires different variants to co-infect a single host), may generate strong background selection.

**Fig. 3:**
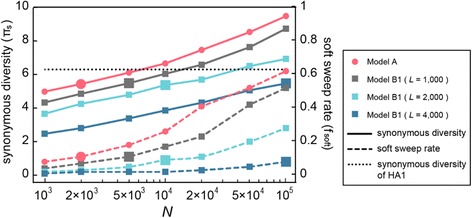
The joint effect of recurrent positive and negative selection with increasing population size. While the rate of adaptive substitutions and selection coefficient (s) are fixed at *k* ≈ 1.3 and *s* = 0.1, synonymous diversity is largest in Model A (*L* = 1,000) and decreases in Model B1 (incorporating background selection) with increasing number of sites under negative selection (*L*_d_ ≈ *L* – 230) with *s*_d_ = 0.1, for a given population size *N*. Simulations with *N* = 2,000 in Model A and (*N*, *L*) = (5,000, 1,000), (10,000, 2,000), or (100,000, 4,000) in Model B1 generate soft selective sweeps at comparable rates (*f*_soft_) (larger markers linked by *dashed lines*), and they also yield similar levels of synonymous diversity (π_s_) (larger markers linked by *solid lines*)

We added background selection to the above model of positive selection (Model A with *s* = 0.1) by imposing negative selection on *L*_d_ (= *L* – *L*_s_ – *L*_b_ = *L*_n_ – *L*_b_) non-beneficial nonsynonymous sites: the wild-type allele at these “deleterious sites” mutate to an alleles with selective disadvantage *s*_d_ (Model B1). With *L*_s_ fixed at 230, the strength of background selection increases as we increase the total length of sequence *L* from 1,000 to 2,000 or 4,000. Simulations with *L* = 2,000 and 4,000 incorporate the additional effect of negative selection operating on the HA2 domain and even on other viral segments, which will affect variation at HA due to limited reassortment between segments. We initially use *s*_d_ = 0.1. (The appropriate value of *s*_d_ will be discussed below.) Again, for each parameter combination of these simulations we adjusted the value of *L*_b_ to achieve *k* ≈ 1.3. As expected, under the same rate of adaptive substitutions, synonymous diversity with background selection is lower than without it (Fig. 3). Compared to the model of positive selection alone but with more frequent sweeps (Model A with *k* ≈ 2.0), variation-reducing effect is slightly weaker with *L* = 1,000 but much stronger with *L* = 2,000 or 4,000 (Fig. 3, Additional file 1: Table S1). Again, larger population size results in higher synonymous diversity. However, when background selection is strong as in the case of *L* = 4,000, π_s_ is still lower than the observed value at a large *N* (= 10^5^). It is possible that much stronger background selection than that generated with *L* = 4,000 is operating in the actual H3N2 population since the combined effect of negative selection over the entire viral genome (~15 kb) can be very large assuming a rate of reassortment that is low relative to the frequency of negative selection.

Addition of background selection also reduces the rate of soft selective sweeps, as expected (Fig. 3). This reduction can be understood as a decrease in the fixation probability of an individual beneficial mutation due to the chance of its occurrence on an unfit (deleterious-mutation-carrying) sequence, the “ruby-in-the-rubbish” process [34] invoked by Koelle and Rasmussen [29]. It is known that, in an asexual population considered here, the proportion of the population with the least deleterious mutation (zero deleterious mutation in most populations under equilibrium), which is approximately equal to the effective population size for neutral polymorphism by background selection as long as *s* is not too large relative to *s*_d_, largely determines this fixation probability [34, 35]. Therefore, we can simply parameterize the rate of soft sweeps by θ_1_ = 2*N*_e1_μ, where *N*_e1_ is the effective population size after taking only background selection into account. (The effective population size after incorporating the effect of selective sweep is denoted *N*_e2_. Then, θ_2_ = 2*N*_e2_μ determines the level of neutral polymorphism.) Using an analytical solution, the equation 8 of Hudson and Kaplan [36] that describes the strength of background selection as a function of mutation rate (cumulative over a non-recombining segment) and selection coefficient, we predict *N*_e1_ of approximately 2,000 with *N* = 5,000 and *L* = 1,000, *N* = 10,000 and *L* = 2,000, or with *N* = 100,000 and *L* = 4,000. As expected, in each of these cases the rate of soft sweep is similar to that of Model A with *N* = 2,000 and these cases also yield very similar levels of synonymous diversity (larger markers in Fig. 3). Such small effective population sizes (*N*_e1_ and *N*_e2_) may be obtained under even larger *N* if background selection is stronger, namely if the effective number of linked deleterious sites is larger and/or the selection coefficient of deleterious alleles is smaller [29].

Next we examine the effect of the strength of negative selection (*s*_d_). Previous studies found that deleterious nonsynonymous mutants are not eliminated rapidly but accumulate in viral lineages bound to go extinct, which correspond to the external branches of the genealogical tree [33, 37]. Such an elevation of deleterious genetic load was quantified as the excess of nonsynonymous-to-synonymous substitution rate (*d*_N_/*d*_S_) in external relative to internal branches. We define ω_i_ and ω_e_ as the average *d*_N_/*d*_S_ in internal and external branches, respectively. The strength of negative selection will affect not only ω_i_ and ω_e_ but also its ratio ω_e_/ω_i_. We therefore used varying *s*_d_ in our simulation and examined its effect on these quantities, in addition to its effect on π_s_ and *f*_soft_. Different values of *s* (for beneficial allele) were also tested as the relative strength of positive and negative selection is expected to affect ω_i_ and ω_e_.

Nonsynonymous and synonymous substitutions were counted from two data sets of H3N2 and the excess of ω_e_ over ω_i_ was observed as expected (Table 1). In simulations under which all non-beneficial nonsynonymous sites are under negative selection with *s*_d_ (Model B1), when *s*_d_ decreases from 0.1 to 0.05 and again to 0.02 while *s* and *L*_n_ are fixed, the values of ω_e_ increase roughly proportionally, among which the best fit to empirical ω_e_ is observed when *s*_d_ = 0.05. On the other hand, ω_i_ increases with decreasing *s*_d_ more dramatically: relative to the empirical value of ω_i_, ω_i_ in simulation is much smaller with *s*_d_ = 0.1 or 0.05 but approximately twice larger with *s*_d_ = 0.02. Although ω_e_/ω_i_ is similar to the observed values when *s*_d_ = 0.02, large values of ω_i_ imply frequent hitchhiking of deleterious mutations to fixations, and this may not be the regime of influenza. This effect of *s*_d_ is consistently observed for all combinations of *s* and *L*_n_. Overall, we failed to find a single value of *s*_d_ that made ω_i_, ω_e_, and ω_e_/ω_i_ fit empirical data simultaneously. However, it might be unrealistic to assume uniform negative selection across all nonsynonymous sites. We thus explored the plausible distribution of *s*_d_ in more realistic models.

**Table 1:** Effect of *s*_d_ on nonsynonymous-to-synonymous substitution ratios for internal (ω_i_) versus external (ω_e_) branches and on π_s_ and *f*_soft_

Dataset or model^a^	Description	*L*	*s*	*s*_d_	ω_i_^a^	ω_e_^a^	ω_e_/ω_i_^a^	π_s_	*f*_soft_	*L*_b_	*N*
Fitch et al. 1997 [37]					0.28	0.40	1.44	6.2	0		
Data from 1987 ~ 1996					0.27	0.35	1.28	6.2	0		
B1	Equal *s*_d_ for all nonsynonymous non-beneficial sites	1,000	0.05	0.02	0.55	0.73	1.36	6.15	0.03	30	2 × 10^3^
				0.05	0.15	0.46	3.11	5.95	0.02	30	
				0.1	0.10	0.26	2.75	6.32	0.04	30	
			0.1	0.02	0.58	0.69	1.21	5.96	0.13	6	10^4^
				0.05	0.15	0.37	2.50	5.87	0.13	6	
				0.1	0.09	0.19	2.13	5.94	0.16	6	
			0.2	0.02	0.56	0.67	1.23	5.89	0.72	2	5 × 10^4^
				0.05	0.16	0.35	2.31	5.53	0.61	2	
				0.1	0.09	0.19	2.25	5.98	0.72	2	
		2,000	0.05	0.02	0.50	0.69	1.41	6.26	0.02	30	10^4^
				0.05	0.11	0.41	3.68	5.75	0.02	35	
				0.1	0.04	0.20	5.42	7.15	0.04	20	
			0.1	0.02	0.51	0.66	1.31	6.18	0.13	6	5 × 10^4^
				0.05	0.12	0.35	3.09	5.77	0.1	7	
				0.1	0.04	0.17	4.22	6.40	0.19	5	
B2	*s*_d_ is either 0.01, 0.02, 0.05, 0.1, or 0.2 (20 % of sites each)	1,000	0.1	0.01 ~ 0.2	0.37	0.44	1.22	6.03	0.14	6	10^4^
		2,000	0.1	0.01 ~ 0.2	0.29	0.41	1.44	6.52	0.16	5	5 × 10^4^
B3	25 % of nonsynonymous non-beneficial sites are neutral (*s*_d_ = 0), another 25 % lethal (*s*_d_ = 1), and the rest with a given value of *s*_d_	1,000	0.1	0.02	0.56	0.59	1.07	6.35	0.19	5	10^4^
				0.05	0.38	0.43	1.16	6.01	0.15	6	
				0.1	0.34	0.35	1.05	6.30	0.21	5	
		2,000	0.1	0.02	0.53	0.59	1.13	6.25	0.17	6	2 × 10^4^
				0.05	0.33	0.42	1.32	5.93	0.14	6	
				0.1	0.29	0.34	1.17	6.31	0.19	5	

^a^For each parameter combination, simulation results were obtained as averages over 300 replicates and their standard errors are within 5 % of the values

First, we tested a model in which *s*_d_ for nonsynonymous sites are either 0.01, 0.02, 0.05, 0.1 or 0.2 (20 % of sites for each) with the mean of 0.076 (Model B2). This led to close agreement with the empirical data. Next we also took into account a previous result in which the fraction of nonsynonymous sites under negative selection is at least 0.7 [5], and in which a substantial proportion of mutations in an RNA virus is lethal [32]. Therefore, we modeled that 25 % of nonsynonymous non-beneficial mutations are neutral, another 25 % lethal, and the other mutations deleterious with equal *s*_d_ (Model B3). This model yielded ω_i_, ω_e_, and ω_e_/ω_i_ quite similar to the empirical values with *L*_n_ = 1770 and *s*_d_ = 0.05 or 0.1. It is highly plausible that other distributions of *s*_d_ would also generate fitting results, while an exhaustive search for such distributions is beyond the scope of this study. A general conclusion we may draw from the above results is that strong negative selection (*s*_d_ = 0.05 ~ 0.1) as assumed in our Models B1 (Fig. 3) is compatible with the excess of deleterious genetic load observed in the external branches of genealogy, whereas weak negative selection results in too large *d*_N_/*d*_S_ in both internal and external branches.

While its impact on *d*_N_/*d*_S_ is notable, varying *s*_d_ does not change π_s_ and *f*_soft_ greatly (Table 1). When simulations with the same *L*_d_ and *s* are compared, π_s_ and *f*_soft_ slightly decrease as *s*_d_ is lowered from 0.1 to 0.05, but increase again as it is further lowered to 0.02. This is probably because interference between numerous sites under selection in a linked segment reduces effective population size so that the effect of negative selection at individual sites becomes weaker, a phenomenon known as Hill-Robertson interference [38]. Therefore, deleterious mutations with small selection coefficient at a linked site do not reduce variation as much as the analytical solution of background selection predicts [39, 40].

### The effect of complex demography

**Fig. 4:**
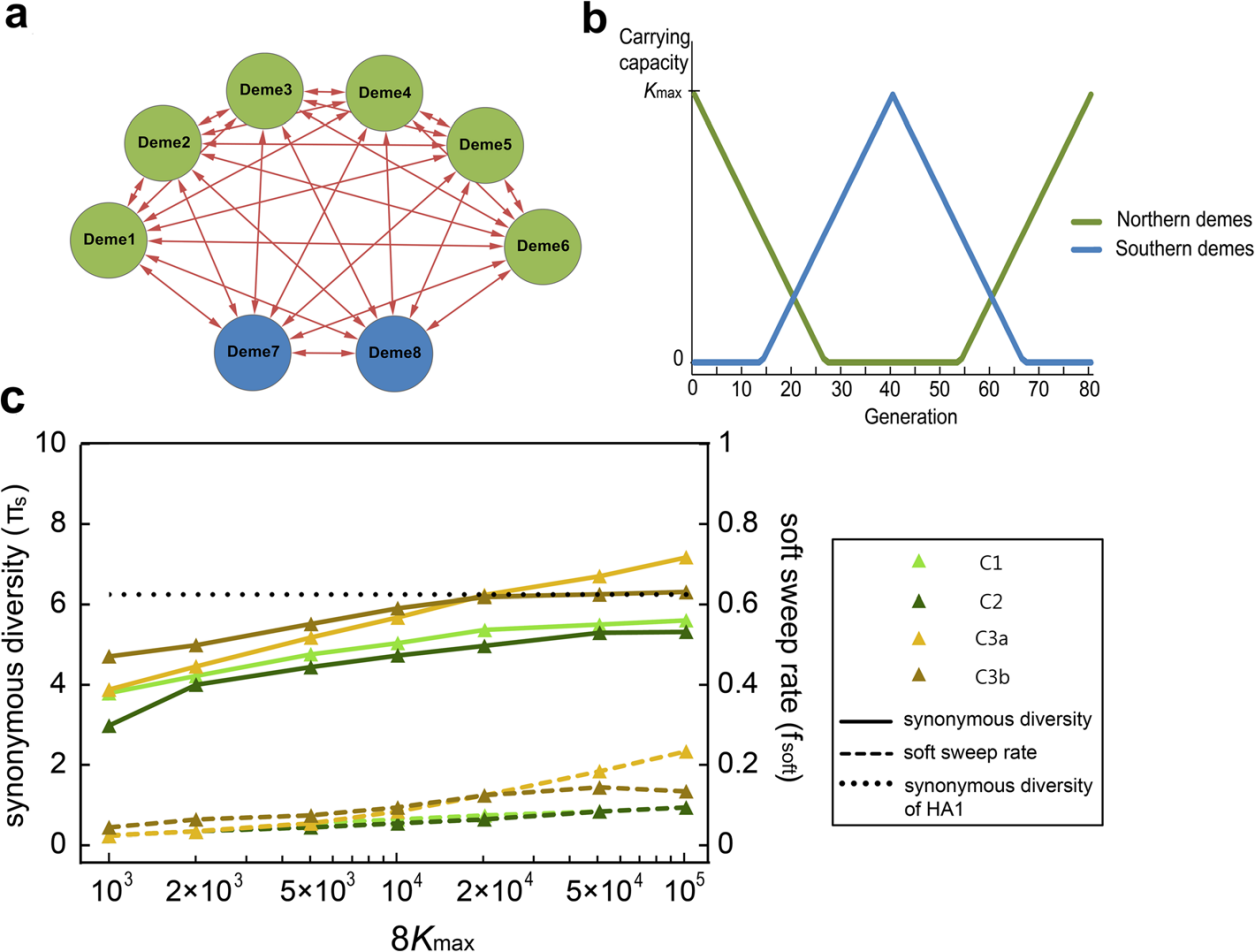
Model of complex demography (metapopulation dynamics) under which recurrent positive selection occurs. **a**. Population is subdivided into six demes in the northern hemisphere (*green*) and two demes in the southern hemisphere (*blue*). Viruses migrate between demes in all directions (*red arrows*). **b**. Seasonal changes in the carrying capacities (*K*) of demes. **c**. Synonymous diversity (π_s_) and soft sweep rate (*f*_soft_) according to population size (*N*) in metapopulation models. C1: rates of migrations in all directions are equal. C2: immigration and emigration rate of a single northern deme is 10 times larger than the other demes. One of the six northern demes does not undergo the extinction-recolonization cycle but maintains *K* = 0.2*K*_max_ (C3a) or 500 (C3b)

As the next candidate for an evolutionary process reducing the effective size of H3N2, we consider its complex demography. Viral reproduction in our simulation model is based on absolute fitness as a function of time-dependent carrying capacity, which allows a straightforward realization of metapopulation (multiple-deme extinction-recolonization) dynamics (see Methods). Different geographic regions over which the global H3N2 epidemics propagate may be modeled as discrete subpopulations (demes). As the yearly cycle of seasonal influenza is largely characterized by alternating epidemics in northern and southern hemispheres [41], we first consider a simple model with six “northern” demes and two “southern” demes (Fig. 4a, b). This model, ignoring the presence of tropical Asian populations where viruses were shown to circulate almost continuously to act as the hub of global viral transmission [8, 25, 26, 42], rather represents a demographic scenario with maximal variation-reducing effect (due to recurrent population bottlenecks, see below). We will later modify this model to take the presence of the tropical population into account.

The carrying capacity of a northern deme reaches its peak (*K*_max_) at the beginning/end of a year (“winter”) but decreases to zero in the middle of a year (“summer”). Conversely, the carrying capacity of a southern deme reaches *K*_max_ in summer and zero in winter. Then, the persistence of the entire metapopulation (i.e. the continuing cycles of extinction-recolonization at a given deme) is critically dependent on the events of migration between northern and southern demes when the carrying capacities at both sides are non-zero. A small number (relative to *K*_max_) of migrant viruses implies recurrent population bottlenecks and thus a reduced effective population size, under which positive selection occurs.

In the simplest model (C1), the rates of migration in all directions are equal (*m*_*ij*_ = *m*_*ji*_). In the second model (C2), in order to test the effect of asymmetric migration rate, a single northern deme which models a region of particularly high viral transmission is receiving and sending 10 times more migrants than other demes. In the third, one of six Northern demes stops the extinction-recolonization cycle and maintains *K* = 0.2*K*_max_ (C3a) or 500 (C3b) throughout the year. This deme corresponds to a tropical Asian population that exhibits continuous viral epidemics. While some estimates of migration rate in H3N2 between actual geographic regions are available [43], it is not clear if those values can be applied to our simplified models of global H3N2 demographic structure. Instead, we considered a migration rate reasonable if it results in the degree of genetic differentiation among demes (*F*_ST_) close to the observation from the actual population (0.18) [26]. In each model and for a given *K*_max_, we could find a unique combination of *L*_b_ and migration rates that yielded both *k* ≈ 1.3 and *F*_ST_ ≈ 0.18 (Additional file 1: Table S3). Note that, to maintain constant *F*_ST_, migration rate needs to decrease as the size of demes increases in Models C1, C2, and C3a, in agreement with the standard island model in which the product of the two parameters determines the level of *F*_ST_. However, in C3b, the relationship between *m* and *K*_max_ for constant *F*_ST_ is more complicated.

**Fig. 5:**
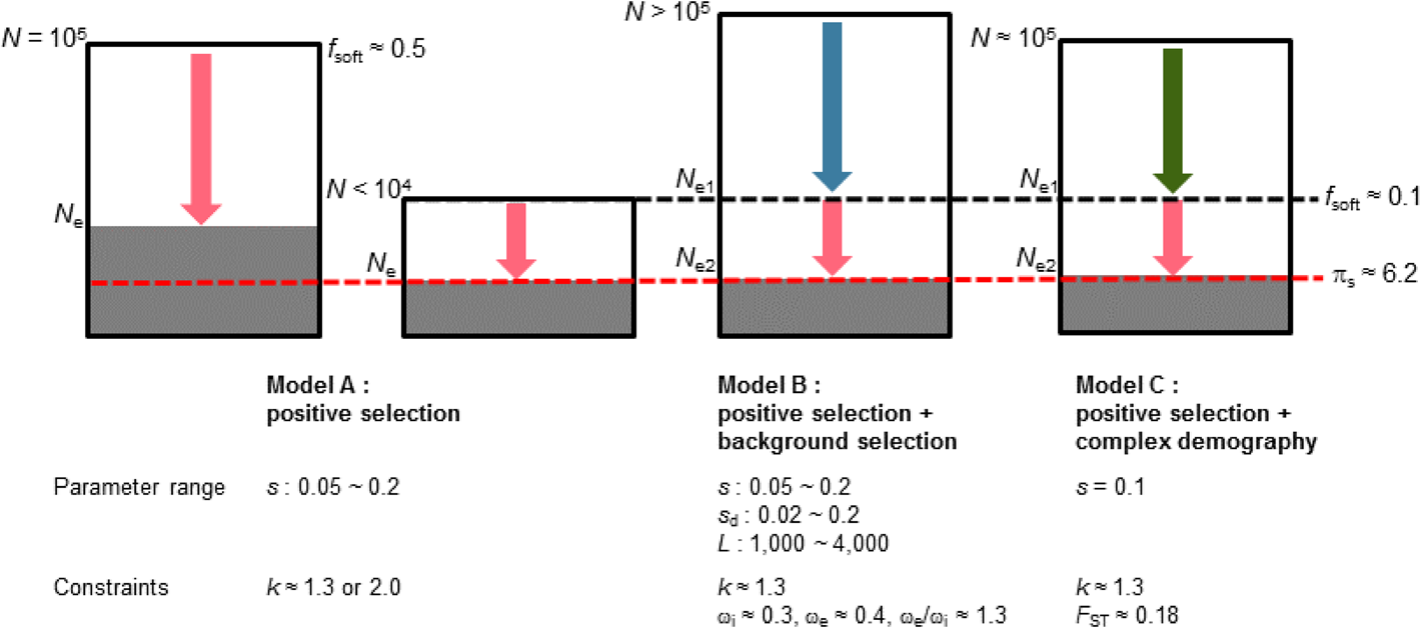
Summary of evolutionary models and the two-step reduction of effective population size. The degrees of reduction in effective population size due to recurrent selective sweeps are shown by *red arrows*. Reductions due to background selection and metapopulation dynamics, when parameters are chosen to maximize the variation-reducing effect (and, at the same time, to satisfy the constraints) of the models, are indicated by *blue* and *green arrows*, respectively. *Black dashed line* represents the census (*N* < 10^4^ in Model A) or effective population size (*N*_e1_ in Model B and C) under which positive selection results in small probability of soft selective sweeps (*f*_soft_ ≈ 0.1). *Red dashed line* marks the effective population size (*N*_e_ in Model A with *N* < 10^4^ and *N*_e2_ in Model B and C) that generate the observed sequence diversity (π_s_ ≈ 6.2). Background selection can generate the observed patterns of soft sweeps and sequence diversity in a population with the largest census size

Simulation results show that, as population size (*K*_max_) increases, π_s_ and *f*_soft_ increases as expected (Fig. 4c). The relationship between π_s_ and *f*_soft_ is similar across different models of metapopulation as well as models of constant-sized population with identical *k* and *s* (1.3 and 0.1, respectively; Fig. 3). This suggests that an identical two-step process of reducing effective population size occurs both with background selection and with metapopulation dynamics. Here, we may define *N*_e1_ as the effective size determined by complex demography alone. Then, positive selection operates under a population with *N*_e1_ to further reduce the effective population size to *N*_e2_. Our result is explained if *N*_e1_ determines *f*_soft_, *N*_e2_ determines π_s_, and *N*_e2_/*N*_e1_ is the marginal effect of recurrent selective sweeps that is invariant regardless whether it occurs jointly with background selection or with metapopulation dynamics as long as *k* and *s* remains the same (Fig. 5).

The effect of reducing effective sizes by metapopulation dynamics is however highly variable depending on the models. In C1 and C2, π_s_ ~ 5.3 was obtained with 8*K*_max_ = 2 × 10^4^ and 10^5^, respectively. Considering that comparable levels of polymorphism were obtained in Model B1 with *N* = 10^4^ and *L* = 2,000 or *N* = 10^5^ and *L* = 4,000 (Fig. 3) and that the census size in C1 and C2 is certainly smaller than 8*K*_max_, we may conclude that the variation-reducing effects of metapopulation dynamics here are similar or slightly weaker than those of background selection. In the presence of a “tropical” deme, despite its small size (*K* = 0.2*K*_max_ in C3a and *K* = 500 in C3b), the effect of metapopulation dynamics greatly diminishes and is weaker than that of background selection in Model B1 with *L* = 4,000, probably because this deme eliminates the effects of recurrent population bottlenecks. These results indicate that the variation-reducing effect of metapopulation dynamics is similar to or weaker than that of background selection, at least with the models and their parameters used in our simulations.

We note that Ferguson et al. [44] also modeled the spatial and seasonal structure of influenza virus by dividing the population into northern and southern demes. Under this model they obtained genealogical structures matching the observed diversity. However, they found only subtle effect of their demographic parameter (ε, the level of seasonal forcing) on the evolutionary dynamics. This is in contrast to our conclusion that the complexity of demography, especially the presence of a tropical deme, is a key determinant of the level of viral genetic diversity. Their result differs probably because they allowed between-season persistence of both northern and southern demes (their “ε” being only around 0.25) and thus precluded the extinction-recolonization dynamics of the metapopulation.

### Relative contributions of evolutionary processes

Given that soft sweeps rarely occurs, *N*_e1_ of H3N2 must not be higher than the order of 10^3^. As given in the Introduction, from the level of sequence polymorphism and branch lengths of the coalescent tree *N*_e2_ is estimated to be on the order of 10^2^ at HA [10]. Therefore, reduction from *N*_e1_ to *N*_e2_ caused by positive selection is on the order of 10 (Fig. 5). On the other hand, the reduction of effective size, from *N* to *N*_e1_, caused by background selection and/or complex demography is likely to be much larger than the order of 10, considering a potentially large census size of H3N2: approximately 0.1 % of the world’s population at any time is infected with influenza viruses [10], among which H3N2 is the major subtype causing flu epidemics [45]. While both background selection and metapopulation dynamics are important determinants of influenza genetic diversity, background selection is a potentially more important factor in reducing the effective population size than complex demography. In influenza H3N2, actual metapopulation dynamics may have a limited impact in reducing effective size due to persistent populations in tropical Asia. On the other hand, background selection may have stronger variation-reducing power than demonstrated in our models, as negative selection against deleterious alleles on the entire influenza genome can affect polymorphism in the HA segment with limited recombination (reassortment). Since we considered one linked segment (HA) and assessed the potential effect of other segments only indirectly by increasing the number of sites subject to deleterious mutations or beneficial mutations, our study is rather conservative in estimating the impact of genome-wide negative and positive selection. It is not easy to extend our model to accommodate genome-wide selective forces and assess their variation-reducing effects quantitatively because the rate of reassortment in H3N2 viruses, which appears to vary for different segments [8, 46], has not been estimated. However, our major conclusion – greater reduction in genetic diversity by background selection than by positive selection – is not likely to change after taking genome-wide selection into account, because negative selection is prevalent in all viral segments whereas recurrent positive selection is known to be limited mostly to HA and NA segments.

## Conclusion

Various models of positive selection have been proposed to explain the limited genetic diversity of HA in H3N2 [5, 7, 10, 18, 44]. Using computer simulations of viral population undergoing recurrent selective sweeps, we have demonstrated that positive selection alone cannot generate the observed level of synonymous diversity and that additional evolutionary forces reducing the effective population size, which also explain the prevalence of hard selective sweeps with antigenic cluster transitions, are needed. The most likely candidates for additional variation-reducing processes are background selection and metapopulation dynamics. Both processes are likely to exert strong effect in the H3N2 viral population, since seasonal influenza viruses are characterized by the absence of intra-segment recombination and by complex metapopulation dynamics. Within the range of parameter values used in our models, which are bounded to match other aspects of H3N2 molecular evolution (see Fig. 5 for summary), we conclude that background selection has potentially more important role in limiting sequence diversity in influenza virus.

Other population genetic factors not considered above might also contribute to reducing the effective population size of H3N2 viruses. For example, the variance of the number of descendant viral copies after replication and transmission, without considering fitness differences, might be very high. Poisson distribution was used in the reproduction step of our simulation model, as it mimics the conventional Wright-Fisher model. We also tried negative binomial distribution with dispersion parameter 0.1, representing a skewed distribution in which a small fraction of viruses produce a large number of descendant copies. However, it had the effect of reducing the effective population size by less than an order of magnitude (data not shown). Further investigation nevertheless is warranted to explore the effect of realistic patterns of viral transmission on the effective size of H3N2 population.

## Methods

### Sequence data

To estimate population genetic parameters, HA sequences of human influenza A/H3N2 sampled between 1968 and 2013 were downloaded from the Influenza Virus Genome Set of National Center for Biotechnology Information (NCBI) [47]. We chose genome set data as it will allow the straightforward comparison of results between segments from analyses planned in the future. However, we confirmed that using all available HA sequences from the database yielded consistent results. After outlier sequences (different from other HA sequences in the same year at more than 100 sites) and sequences that contain symbols other than A, C, G, and T were discarded, a total of 3,888 sequences were used for analyses. To obtain synonymous diversity of the HA1 domain, mean pairwise synonymous differences among sequences sampled within the same year was calculated using DnaSP5 and averaged over the period from 1968 to 2013. Substitution rate was estimated from the divergence between sequences in 1968 and in 2013 at synonymous sites divided by the number of sites and by 45 years.

### Reconstruction of genealogies and detecting soft sweeps

To investigate the mode of selective sweeps of antigenic-cluster-changing substitutions reported in [19], we reconstructed the genealogical tree of HA1 and visually observed whether multiple copies of the same beneficial allele arose independently from different ancestral backgrounds and went to fixation together, meaning that in the first year in which the frequency of the derived allele reached 1.0 these different clones of beneficial allele coexisted. Each substitution was examined on the genealogy generated from all sequences available at the Influenza Virus Resource [47] so that we would not miss minor lineages arising from recurrent mutations. Maximum likelihood trees were constructed using MEGA6 [48] under the HKY substitution model and four discrete categories of gamma distributed plus invariant sites (G + I) model.

### The range of selection coefficient (*s*) for a beneficial allele

To find the reasonable range of the strength of positive selection acting on H3N2 viruses, we inferred selection coefficient (*s*) from how rapidly the frequencies of antigen-changing mutations increase. Namely, we fit an allele frequency change to

$$ t\approx \frac{1}{s} \ln \left({u}_t/{u}_0\right) $$

[49] where *u*_0_ and *u*_*t*_ are the ratio of derived (*q*) to ancestral (*p*) allele frequency in time 0 and *t* (in generation), respectively. For each trajectory, we chose two time points approximately corresponding to *q* = 0.1 and 0.9. Since sampling was sparse and information for the month of collection was usually absent before the 1990s, we estimated *s* only from mutations that occurred later than 1990 out of 17 mutations in Koel et al. [19]. The frequencies of antigenic changing mutations through time were obtained from sliding windows of 6 months with a step size of 1 month. Time in months estimated to be taken for frequency to increase from 0.1 to 0.9 was then converted to time in generations. Estimated *s* ranged between 0.05 and 0.11. Changing window and step sizes did not affect our estimation of *s*. In the trajectories of mutations from which relatively low selection coefficients were estimated, allele frequencies were often observed to fluctuate even after they reach higher than 0.3 and maintained intermediate values for several months. This is probably due to clonal interference and would lead to underestimating their fitness effects. We confirmed that selection coefficients are underestimated from frequency trajectories generated in our simulation (Model A), where the rate of adaptive substitutions is sufficiently high enough to cause clonal interference. Considering these results, we choose *s* = 0.1 as a reasonable parameter for our further investigation.

### Simulation

A genetic-demographic model for viral evolution was built to investigate the patterns of sequence variation under various models of selection and demography, including recurrent selective sweeps in a constant-sized population with or without background selection, or in multiple demes under cycles of colonization-extinction (metapopulation dynamics). In our model, an individual virus is represented by a non-recombining sequence of *L* loci (sites). While this structure mainly models the nucleotide sequence of the HA1 domain of the hemagglutinin gene, each locus carries allele “0” or “1” for simplicity. (As we reconstruct influenza sequence polymorphism with per-nucleotide sequence diversity on the order of 0.01, which is effectively in the domain of an infinite sites model, the use of bi-allelic sites would be justified.) Viruses are assumed to form either a metapopulation divided into *D* (>1) subpopulations (demes) or a single panmictic population (thus *D* = 1). Subpopulations correspond to different geographic populations of influenza virus, connected by migration. While the actual transmission/reproduction cycles of viruses must be continuous and overlapping in time, we modeled it to occur in discrete time steps. As we consider the dynamics of seasonal influenza, an important unit of time is 1 year. One year is divided into 80 viral “generations”.

A reproductive cycle in one generation is divided into steps of replication, mutation, and migration. The number of viruses before reproduction in deme *j* (=1,…, *D*) at a given generation *t* is given by *N*_*j*_ = *N*_*j*_(*t*). Each of these *N*_*j*_ viruses is subject to replication: a virus of sequence ***S*** leaves *V* descendants to generation *t* + 1, where *V* is Poisson distributed with mean *w*(***S***, *j*, *t*), which is the sequence-dependent absolute fitness of a virus:

1 $$ w\left(\boldsymbol{S},j,t\right)={w}_{\boldsymbol{S}}\frac{1+g}{1+g{N}_j/K} $$

where *w*_***S***_ is the relative fitness of the parental sequence ***S*** (defined below), *g* is the intrinsic growth (multiplication) rate of virus, and *K* = *K*(*j*, *t*) is the carrying capacity of deme *j* at generation *t*. We used Poisson distribution as it approximates the Wright-Fisher model of reproduction and thus makes the effective population size quantified here comparable to other studies. In the model of a single panmictic population *K* does not change over time and is equal to *N*, the population size. In the model of metapopulation dynamics, *K* describes the seasonal demographic cycle of a viral subpopulation. Different demes may peak in size at different times of year, thus mimicking the temporal shift of influenza epidemics in the global geographic regions along the latitude. In a simplified model of their geographic distribution, six demes are placed in the northern hemisphere and two other demes in the southern hemisphere. A northern deme with its peak in “winter” is modeled by *K*(*j*, *t*) = *K*_*max*_(1 – *t*/26.4) for *t* = 0 to 26, 0 for *t* = 27 to 53, and *K*_*max*_(*t*/26.4 – 2) for *t* = 54 to 79. With a high intrinsic growth rate (we used *g* = 1 throughout the simulation), *N*_*j*_ closely follows *K*, once new migrants to deme *j* start reproducing after its *K* increases above zero. The deme becomes extinct when *K* becomes zero. After replication, bi-directional mutation (“0” to/from “1”) occurs with probability μ per site.

In the next step of migration (only in the model of metapopulation dynamics), *M*_*ij*_ viruses are randomly chosen from deme *i* and are sent to deme *j. M*_*ij*_ is Poisson distributed with mean *m*_*ij*_*N*_*i*_*’*, where *m*_*ij*_ is the time- and sequence-independent probability that a virus in deme *i* migrates to deme *j* per generation and *N*_*i*_*’* is the number of viruses in deme *i* after viral replication and mutation. Namely, the number of migrants is proportional to the size of source population. After migration occurs between all pairs of demes, the reproduction to generation *t* + 1 is completed, with *N*_*j*_ = *N*_*j*_ ' + ∑_*i* ≠ *j*_(*M*_*ij*_ − *M*_*ji*_).

Of *L* sites in the viral sequence, *L*_s_ sites correspond to “synonymous” sites that mutate to neutral alleles. There are *L*_b_ “beneficial” sites among *L*_n_ = *L* - *L*_s_ “nonsynonymous” sites. On beneficial sites a new mutation is assumed to increase the relative fitness of the viral sequence by *s*. The rate of mutation to such a beneficial allele is assumed to be μ_b_ = μ/3, considering that different nonsynonymous nucleotide changes at a site may produce different amino acid changes while only one of them is likely to be beneficial. At these sites, mutation is uni-directional, from the ancestral allele (“0”) to the derived allele (“1”). If the derived allele reaches fixation, all its copies reset to the ancestral allele in order to maintain *L*_b_ and thus the rate for adaptive substitutions (*k*) through time. On the other *L*_d_ = *L* - *L*_s_ - *L*_b_ nonsynonymous sites, bi-directional mutations with rate μ are either neutral (models of positive selection only) or deleterious (models of both positive selection and negative selection). The (marginal) relative fitness of a virus carrying a deleterious allele is given by 1 - *s*_d_. Then, the relative fitness of sequence ***S*** is given by

2 $$ {w}_{\boldsymbol{S}}={\displaystyle {\prod}_{i=1}^{L_{\mathrm{b}}+{L}_{\mathrm{d}}}{w}_i/\overline{w}} $$

where *w*_*i*_ is the relative fitness of an allele on the *i*th allele, which is 1 if it is ancestral, 1 + *s* if beneficial, and 1 - *s*_d_ if deleterious, and $\overline{w}$ is the mean fitness of sequences in the whole population.

For each model, we ran simulations increasing *N* from 10^3^ to 10^5^. For each parameter set, the simulation was repeated 1,000 times except for *N* > 10^4^ in background selection, for which 300 replicates were made. In each replicate, first 40,000 generations were removed as burn-in and from next 10 years (800 generations) 40 sequences were randomly sampled every 8 generations. Synonymous diversity is estimated from 230 synonymous sites by calculating pairwise diversity among sequences sampled in the same year and then averaged over years and replicates.

### Detecting soft sweeps from the simulation

When the derived allele at a beneficial site (identical by state) became fixed, we examined the mode of a selective sweep from 40 sequences sampled prior to or at the generation of fixation. We counted the numbers of clones, which are defined as groups of sequences descending from independent beneficial mutations and calculated

$$ H=1-{\displaystyle {\sum}_{i=1}^n{p}_i^2} $$

where *p*_*i*_ is the frequency of *i*th clone. We define that a selective sweep is soft if *H* > 0.1.

### Ratio of nonsynonymous to synonymous substitutions (*d*_N_/*d*_S_)

We calculated *d*_N_/*d*_S_ on the individual branches of genealogical trees obtained from two different HA1 datasets of influenza H3N2. One is dataset analyzed by Fitch et al. [37] and the other is 10 replicate sets of viral samples over which *d*_N_/*d*_S_ will be averaged. For the latter, sequences of each set were sampled from all available data in NCBI collected between 1987 and 1996. If the number of available sequences of a year was 26 or less, entire sequences were used; otherwise, 26 sequences were randomly sampled per year so that the total number of sequences in a set matches that of Fitch et al. [37] (*n* = 254). Neighbor-joining trees were constructed from each dataset with BreakTies RANDOM option and states of internal nodes were inferred using maximum parsimony with ACCTRAN option using PAUP 4 test version [50]. We estimate *d*_N_/*d*_S_ of internal (external) branches by

$$ \begin{array}{l}\upomega =\frac{{\displaystyle {\sum}_i}{D}_i^{\left(\mathrm{N}\right)}}{{\displaystyle {\sum}_i}{D}_i^{\left(\mathrm{S}\right)}}/\frac{{\displaystyle {\sum}_i}{L}_i^{\left(\mathrm{N}\right)}}{{\displaystyle {\sum}_i}{L}_i^{\left(\mathrm{S}\right)}}\\ {}\end{array} $$

where *D*_*i*_^(S)^ and *D*_*i*_^(N)^ are the number of synonymous and nonsynonymous differences in the *i*^th^ internal (external) branch, and *L*_*i*_^(S)^ and *L*_*i*_^(N)^ are the numbers of synonymous and nonsynonymous sites calculated for branch *i* according to Nei-Gojobori method.

We calculate *d*_N_/*d*_S_ from our simulation in a similar way. For each combination of simulation parameters (*s*_d_, *s*, and *L*_n_), we adjusted *N* and the number of beneficial sites (*L*_b_) to yield a rate of selective sweeps (*k*) close to 1.3, ran 300 replicates, and kept sequences from last 10 simulation years. From each replicate 26 sequences were sampled per year, making 300 sets of 260 sequences, over which *d*_N_/*d*_S_ was averaged.

## Additional file

Additional file 1: Table S1. Parameters and results of positive selection alone model (Model A). **Table S2.** Parameters and results of background selection model (Model B1). **Table S3.** Parameters and results of complex demography models (Model C). (DOCX 26 kb)
