# Supplementary material for: Population genetic processes affecting the mode of selective sweeps and effective population size in influenza virus H3N2
Source: BMC Evol Biol. 2016 Aug 3;16:156. doi: 10.1186/s12862-016-0727-8 (PMC4972962; doi:10.1186/s12862-016-0727-8)
Supplement: Additional file 1: Table S1. — Parameters and results of positive selection alone model (Model A). Table S2. Parameters and results of background selection model (Model B1). Table S3. Parameters and results of complex demography models (Model C). (DOCX 26 kb) [file 12862_2016_727_MOESM1_ESM.docx]

Table S1. Parameters and results of positive selection alone model (Model A)

| *k* | *s* |  | *N* | | | | | | |
| --- | --- | --- | --- | --- | --- | --- | --- | --- | --- |
|  |  |  | 10^3^ | 2×10^3^ | 5×10^3^ | 10^4^ | 2×10^4^ | 5×10^4^ | 10^5^ |
| 1.3 | 0.1 | *L*_b_ | 10 | 8 | 6 | 5 | 4 | 4 | 4 |
|  |  | *k* | 1.27 | 1.34 | 1.36 | 1.34 | 1.26 | 1.33 | 1.36 |
|  |  | π_s_ | 4.88 | 5.33 | 6.01 | 6.56 | 7.36 | 8.43 | 9.38 |
|  |  | *f*_soft_ | 0.07 | 0.10 | 0.17 | 0.25 | 0.40 | 0.51 | 0.61 |
|  | 0.05 | *L*_b_ | 25 | 19 | 14 | 12 | 10 | 9 | 8 |
|  |  | *k* | 1.30 | 1.31 | 1.30 | 1.32 | 1.28 | 1.30 | 1.27 |
|  |  | π_s_ | 6.91 | 7.32 | 8.34 | 8.95 | 9.80 | 10.69 | 11.64 |
|  |  | *f*_soft_ | 0.04 | 0.06 | 0.09 | 0.11 | 0.14 | 0.20 | 0.27 |
|  | 0.2 | *L*_b_ | 4 | 3 | 2 | 2 | 2 | 2 | 2 |
|  |  | *k* | 1.22 | 1.27 | 1.22 | 1.33 | 1.39 | 1.40 | 1.39 |
|  |  | π_s_ | 3.86 | 4.06 | 4.56 | 4.97 | 5.75 | 7.63 | 9.86 |
|  |  | *f*_soft_ | 0.09 | 0.16 | 0.36 | 0.52 | 0.68 | 0.85 | 0.91 |
|  | Uniformly distributed from 0.01 to 0.1 | *L*_b_ | 22 | 16 | 12 | 11 | 9 | 8 | 7 |
|  |  | *k* | 1.29 | 1.27 | 1.27 | 1.33 | 1.29 | 1.31 | 1.26 |
|  |  | π_s_ | 5.45 | 6.09 | 6.86 | 7.41 | 8.19 | 9.29 | 10.60 |
|  |  | *f*_soft_ | 0.06 | 0.09 | 0.16 | 0.21 | 0.31 | 0.43 | 0.55 |
|  | 0.01 (10× generations per year: 800 generations per year,  0.1× μ: μ = 10^-5^) | *L*_b_ | 40 | 24 | 13 | 9 | 6 | 5 | 4 |
|  |  | *k* | 1.32 | 1.32 | 1.31 | 1.32 | 1.24 | 1.32 | 1.26 |
|  |  | π_s_ | 2.93 | 3.58 | 4.31 | 4.83 | 5.37 | 5.94 | 6.59 |
|  |  | *f*_soft_ | 0.01 | 0.02 | 0.05 | 0.08 | 0.13 | 0.21 | 0.32 |
| 2.0 | 0.1 | *L*_b_ | 22 | 17 | 13 | 10 | 9 | 8 | 7 |
|  |  | *k* | 1.97 | 2.00 | 2.06 | 1.98 | 2.01 | 2.04 | 1.98 |
|  |  | π_s_ | 4.45 | 4.74 | 5.23 | 5.76 | 6.04 | 6.63 | 7.15 |
|  |  | *f*_soft_ | 0.04 | 0.06 | 0.09 | 0.12 | 0.15 | 0.21 | 0.29 |

Table S2. Parameters and results of background selection model (Model B1), which incorporates background selection to Model A with *k* = 1.3 and *s* = 0.1

| *L* |  | *N* | | | | | | |
| --- | --- | --- | --- | --- | --- | --- | --- | --- |
|  |  | 10^3^ | 2×10^3^ | 5×10^3^ | 10^4^ | 2×10^4^ | 5×10^4^ | 10^5^ |
| 1,000 | *L*_b_ | 17 | 12 | 8 | 6 | 5 | 4 | 4 |
|  | *k* | 1.33 | 1.36 | 1.37 | 1.32 | 1.30 | 1.25 | 1.29 |
|  | π_s_ | 4.23 | 4.75 | 5.38 | 5.94 | 6.48 | 7.53 | 8.63 |
|  | *f*_soft_ | 0.03 | 0.06 | 0.1 | 0.16 | 0.22 | 0.41 | 0.51 |
| 2,000 | *L*_b_ | 32 | 20 | 12 | 8 | 7 | 5 | 5 |
|  | *k* | 1.28 | 1.26 | 1.29 | 1.23 | 1.33 | 1.26 | 1.36 |
|  | π_s_ | 3.56 | 4.15 | 4.69 | 5.27 | 5.59 | 6.40 | 6.83 |
|  | *f*_soft_ | 0.01 | 0.02 | 0.04 | 0.08 | 0.10 | 0.19 | 0.27 |
| 4,000 | *L*_b_ | 78 | 60 | 39 | 28 | 19 | 11 | 8 |
|  | *k* | 1.28 | 1.29 | 1.29 | 1.32 | 1.32 | 1.28 | 1.27 |
|  | π_s_ | 2.37 | 2.70 | 3.28 | 3.76 | 4.22 | 4.96 | 5.37 |
|  | *f*_soft_ | 0.00 | 0.01 | 0.01 | 0.01 | 0.02 | 0.04 | 0.07 |

Table S3. Parameters and results of complex demography models (Model C), which incorporate metapopulation dynamics to Model A with *k* = 1.3 and *s* = 0.1

| Model | Description |  | 8*K*_max_ | | | | | | |
| --- | --- | --- | --- | --- | --- | --- | --- | --- | --- |
|  |  |  | 10^3^ | 2×10^3^ | 5×10^3^ | 10^4^ | 2×10^4^ | 5×10^4^ | 10^5^ |
| C1 | Equal migration rate among 8 demes | *L*_b_ | 40 | 30 | 22 | 18 | 14 | 13 | 12 |
|  |  | *m* | 3.6×10^-3^ | 1.8×10^-3^ | 9×10^-4^ | 5×10^-4^ | 3×10^-4^ | 1.2×10^-4^ | 6.5×10^-5^ |
|  |  | *k* | 1.27 | 1.28 | 1.32 | 1.32 | 1.28 | 1.30 | 1.3 |
|  |  | *F*_ST_ | 0.17 | 0.18 | 0.18 | 0.18 | 0.17 | 0.19 | 0.19 |
|  |  | π_s_ | 3.75 | 4.18 | 4.72 | 5.00 | 5.33 | 5.46 | 5.57 |
|  |  | *f*_soft_ | 0.02 | 0.03 | 0.05 | 0.06 | 0.07 | 0.08 | 0.09 |
| C2 | One deme with 10× *m* | *L*_b_ | 40 | 31 | 22 | 17 | 14 | 11 | 11 |
|  |  | *m* | 1.6×10^-3^ | 8.0×10^-3^ | 3.5×10^-4^ | 2.0×10^-4^ | 1.1×10^-4^ | 6.0×10^-5^ | 3.0×10^-5^ |
|  |  | *k* | 1.28 | 1.32 | 1.29 | 1.28 | 1.26 | 1.28 | 1.33 |
|  |  | *F*_ST_ | 0.17 | 0.17 | 0.18 | 0.18 | 0.18 | 0.17 | 0.18 |
|  |  | π_s_ | 2.94 | 3.96 | 4.40 | 4.69 | 4.93 | 5.26 | 5.28 |
|  |  | *f*_soft_ | 0.02 | 0.03 | 0.04 | 0.05 | 0.06 | 0.08 | 0.09 |
| C3a | One deme with constant *K* = 0.2*K*_max_ | *L*_b_ | 39 | 28 | 19 | 15 | 11 | 9 | 7 |
|  |  | *m* | 3.5×10^-3^ | 2×10^-3^ | 9.2×10^-4^ | 5.5×10^-4^ | 3.3×10^-4^ | 1.5×10^-4^ | 8×10^-5^ |
|  |  | *k* | 1.30 | 1.32 | 1.31 | 1.32 | 1.27 | 1.33 | 1.29 |
|  |  | *F*_ST_ | 0.17 | 0.18 | 0.18 | 0.18 | 0.18 | 0.18 | 0.18 |
|  |  | π_s_ | 3.84 | 4.42 | 5.14 | 5.64 | 6.19 | 6.66 | 7.13 |
|  |  | *f*_soft_ | 0.02 | 0.03 | 0.05 | 0.08 | 0.12 | 0.18 | 0.23 |
| C3b | One deme with constant *K* = 500 | *L*_b_ | 16 | 15 | 14 | 12 | 11 | 11 | 11 |
|  |  | *m* | 1.6×10^-4^ | 5×10^-4^ | 6×10^-4^ | 5×10^-4^ | 3.3×10^-4^ | 1.5×10^-4^ | 8×10^-5^ |
|  |  | *k* | 1.33 | 1.30 | 1.31 | 1.28 | 1.27 | 1.32 | 1.33 |
|  |  | *F*_ST_ | 0.17 | 0.18 | 0.18 | 0.18 | 0.18 | 0.18 | 0.18 |
|  |  | π_s_ | 4.67 | 4.95 | 5.48 | 5.87 | 6.15 | 6.21 | 6.27 |
|  |  | *f*_soft_ | 0.04 | 0.06 | 0.07 | 0.09 | 0.12 | 0.14 | 0.13 |
